# Decontamination Effect of Hypochlorous Acid Dry Mist on Selected Bacteria, Viruses, Spores, and Fungi as Well as on Components of Electronic Systems

**DOI:** 10.3390/ijms25137198

**Published:** 2024-06-29

**Authors:** Barbara Nasiłowska, Maksymilian Włodarski, Miron Kaliszewski, Zdzisław Bogdanowicz, Łukasz Krzowski, Krzysztof Kopczyński, Grzegorz Witkowski, Agnieszka Czeczott-Urban, Aneta Bombalska, Magdalena Urbańska, Katarzyna Garbat, Aleksandra Sowińska, Marta Kutwin, Wojciech Koperski, Ryszard Woźniak, Zygmunt Mierczyk

**Affiliations:** 1 Institute of Optoelectronics, Military University of Technology, gen. S. Kaliskiego 2, 00-908 Warsaw, Polandmiron.kaliszewski@wat.edu.pl (M.K.); lukasz.krzowski@wat.edu.pl (Ł.K.); krzysztof.kopczynski@wat.edu.pl (K.K.); aneta.bombalska@wat.edu.pl (A.B.); aleksandra.sowinska@student.wat.edu.pl (A.S.); zygmunt.mierczyk@wat.edu.pl (Z.M.); 2 Faculty of Mechanical Engineering, Military University of Technology, gen. S. Kaliskiego 2, 00-908 Warsaw, Poland; zdzislaw.bogdanowicz@wat.edu.pl; 3 BioMedAqua Sp. z o.o., 39-200 Dębica, Poland; g.witkowski@biomedaqua.eu; 4 Polcargo International Sp. z o.o., 71-899 Szczecin, Poland; a.czeczott@polcargo.pl; 5 Faculty of Advanced Technologies and Chemistry, Military University of Technology, gen. S. Kaliskiego 2, 00-908 Warsaw, Poland; magdalena.urbanska@wat.edu.pl (M.U.); katarzyna.garbat@wat.edu.pl (K.G.); 6 Department of Nanobiotechnology, Institute of Biology, Warsaw University of Life Sciences, Ciszewskiego 8, 02-786 Warsaw, Poland; marta_kutwin@sggw.edu.pl; 7 Institute of Armament Technology, Military University of Technology, gen. S. Kaliskiego 2, 00-908 Warsaw, Polandryszard.wozniak@wat.edu.pl (R.W.)

**Keywords:** hypochlorous acid, decontamination, disinfection, pulse oximeter, Raspberry Pi Zero, dry fog

## Abstract

This publication presents the effect of hypochlorous acid dry mist as a disinfectant on selected bacteria, viruses, spores, and fungi as well as on portable Microlife OXY 300 finger pulse oximeters and electronic systems of Raspberry Pi Zero microcomputers. The impact of hypochlorous acid on microbiological agents was assessed at concentrations of 300, 500, and 2000 ppm of HClO according to PN-EN 17272 (Variant I). Studies of the impact of hypochlorous acid fog on electronic components were carried out in an aerosol chamber at concentrations of 500 ppm and 2000 ppm according to two models consisting of 30 (Variant II) and 90 fogging cycles (Variant III). Each cycle included the process of generating a dry mist of hypochlorous acid (25 mL/m^3^), decontamination of the test elements, as well as cleaning the chamber of the disinfectant agent. The exposure of the materials examined on hypochlorous acid dry mist in all variants resulted in a decrease in the number of viruses, bacteria, spores, and fungi tested. In addition, the research showed that in the variants of hypochlorous acid fogging cycles analyzed, no changes in performance parameters and no penetration of dry fog of hypochlorous acid into the interior of the tested medical devices and electronic systems were observed.

## 1. Introduction

Currently, emerging biological threats, not only in hospitals but also pandemics, such as SARS-CoV-2, have hastened the search for safe disinfection methods that will lead to the neutralization of the pathogenic factor and, at the same time, do not affect medical devices located at the place of operation disinfection.

Regarding anti-epidemic activities, an essential method of limiting the spread of a pathogenic factor is to cut off the path of its spread, which is mainly through the air [1]. The risk of infection increases in poorly ventilated rooms. Biological aerosol containing the SARS-CoV-2 virus may also fall onto nearby surfaces [2]. Kasloff and his team [3] showed that the virus persists on smooth (non-porous) surfaces for up to 21 days. However, Lewis [4] observed in his research that the SARS-CoV-2 virus can be transmitted indirectly through surfaces and thus lead to the spread of the disease.

Healthcare facilities, especially intensive care units, are critical because they are where the concentration of the SARS-CoV-2 virus, MRSA (Methicillin-resistant *Staphylococcus aureus*), VRE (Vancomycin-resistant enterococci), *Clostridioides difficile*, and *Acinetobacter baumannii* strains is highest [5,6].

The very structure of the SARS-CoV-2 virus particle makes it susceptible to commonly used disinfectants, including alcohol-based (with a concentration of at least 70%), hypochlorite, formaldehyde, or hydrogen peroxide [7].

However, decontamination is mainly practiced in contact disinfection, for example, by spraying a disinfectant and wiping a potentially contaminated surface. Another disinfection method that significantly facilitates the process in hard-to-reach areas (such as hospital rooms or ambulances) is the fumigation method using, for example, hydrogen peroxide, formaldehyde, and ozone [8].

Current disinfection methods used in medical centers, including hospitals, are limited to washing surfaces and fumigating, mainly using hydrogen peroxide. Such processes can be used only when patients are not at the place of disinfection and only a few hours after the process to remove the agent altogether. Moreover, the agent used for fumigation must be appropriately safe for medical equipment located in patient rooms.

The selection of an appropriate disinfectant should consider antimicrobial effectiveness, patient and medical staff safety, and lack of negative impact on medical equipment and apparatus.

From the point of view of the above disinfectant properties, one interesting agent is hypochlorous acid (HClO), a natural endogenous substance found in mammals. This substance participates in the non-specific immune response and the reaction of the phagocytic system and thus has antimicrobial properties that act against microorganisms, including bacteria and viruses. Therefore, it is commonly used in open treatment [8].

Hypochlorous acid at a concentration of 200 ppm effectively disinfects surfaces containing noroviruses and other enteric viruses during a contact time of 1 min [5]. Interestingly, after 10-fold dilution, HClO solutions are still effective in disinfecting surfaces with viruses, but contact must be extended to 10 min [5,9].

The mechanism of action of hypochlorous acid on microorganisms is complex and includes many processes occurring both inside cells and at the level of the cell membrane. Due to their moderate molecular size (comparable to water), HClO molecules can penetrate the lipid double layer of the cell membrane by passive diffusion. By damaging the cell membrane, they quickly enter the microbial cell, causing a loss of cellular integrity, thus accelerating the rate of deactivation [10].

Moreover, hypochlorous acid has a strong oxidizing effect, thanks to which it denatures and aggregates proteins [11]. Penetrating the inside of the microorganism causes various damage at the cellular level, including disorganizing the structure of proteins that are key to cell function. This leads to the disruption of life processes and, ultimately, death [12,13,14,15,16]. The lethal effect of HClO is due to the oxidation of sulfhydryl groups (SH), essential enzymes, and antioxidants, and harmful effects on DNA synthesis [13].

Although most currently used disinfectants adversely affect the structure of materials used to build medical devices, causing corrosion, accelerated aging, discoloration, and damage to electronic systems, the effectiveness of disinfection is typically only checked in biological areas. Therefore, this experiment aimed to complement the current state of knowledge regarding the impact of hypochlorous acid on selected strains of bacteria, viruses, and fungi, as well as the impact of dry hypochlorous acid mist on the casing and internal elements of portable Microlife OXY 300 finger pulse oximeters and Raspberry Pi Zero microcomputers, which simulate the electronic systems of medical equipment.

## 2. Results

### 2.1. Results of Physicochemical Tests of Hypochlorous Acid 

Table 1 shows the results of hypochlorous acid surface tension and density tests at concentrations of 200, 300, 400, 500, and 2000 ppm. The tests were conducted at temperatures of 20 and 22 °C. The test results for HClO concentrations of 200–500 ppm did not differ. Slight differences of 1% were observed for hypochlorous acid concentrations of 2000 ppm. The results testify to the stability of surface tension and density of hypochlorous acid in the concentration range of 200 ± 2000 ppm.

**Figure 1:**
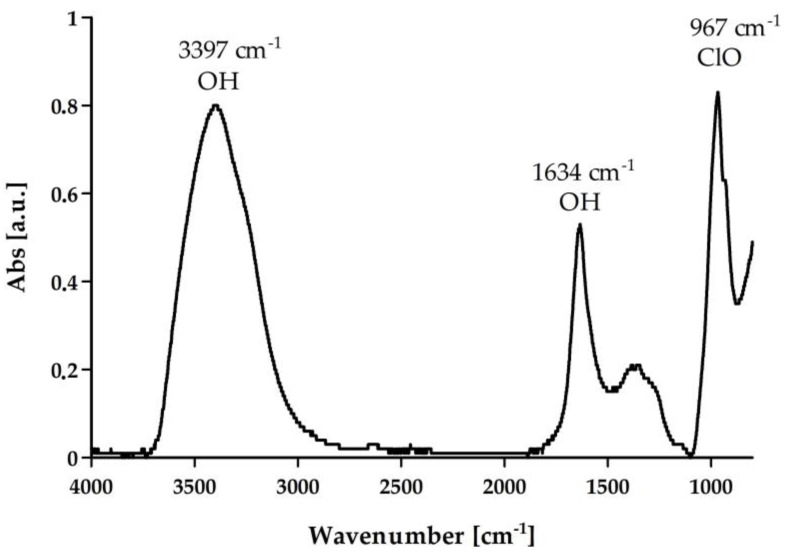
FTIR spectrum of hypochlorous acid.

FTIR studies show typical peaks for hypochlorous acid. Wide absorption bands were recorded for OH groups at 3397 and 1634 cm^−1^ and OCl groups at 967 cm^−1^ (Figure 1) [17].

### 2.2. Hypochlorous Acid Dry Mist Test Results 

The measurements of the free chlorine level performed in the hypochlorous acid dry mist tester at concentrations of 300 ppm, 500 ppm, and 2000 ppm are presented in Table 2. The tests were performed 2, 5, and 10 min after the end of the dry fog generation of hypochlorous acid. The level of free chlorine at 300 and 500 ppm oscillated at a similar level of 0.10–0.20 ppm of Cl. On the other hand, the largest increase in free chlorine was observed for the concentration of HClO 2000 ppm, which was 1.40–1.55 ppm of Cl.

Table 3 The table shows the aerosol particle size distribution after 4.5 min of hypochlorous acid dry mist generation in the chamber at concentrations of 300 ppm. Comparing the size of the particles present before the tests, we can see a clear increase in microparticles, indicating the presence of dry fog.

The room temperature during the dry fog generation was 21 ÷ 23 °C and the air humidity was 95–98%. 

### 2.3. Microbiological Tests 

The analysis of the effectiveness of disinfection by the first cycle of dry fogging with HClO at concentrations of 300 ppm, 500 ppm, and 2000 ppm (Table 4) was carried out against microorganisms indicated in [18] and additional strains of microorganisms discussed in Section 4.3.3. Treatment and measurement repetition were based on the recommendations of the standard PN-EN 17272:2020.

For bacteria specified in the PN-EN 17272 standard and for additional bacterial strains, the required reduction of ≥5 lg was achieved at a concentration of 300 ppm of HClO. HClO dry mist disinfection has bactericidal properties for both Gram-negative and Gram-positive bacteria. In addition, the results of microorganism culture at the level of ≥4 lg reduction for Mycobacterium terrae and Mycobacterium avium and ≥5 lg for Clostridioides dofficile with the use of dry mist of hypochlorous acid at a concentration of 500 ppm indicate a bactericidal effect against mycobacteria. 

On the other hand, in the case of Bacillus subtili spores and Aspergillus brasilienis, a reduction level of ≥4 lg was achieved after using a concentration of 2000 ppm of HClO.

The reduction rate required in [18] for normative viruses of ≥4 lg was already achieved for the modified vaccinia virus Ankara (MVA), as a surrogate for SARS-CoV-2, with the use of hypochlorous acid at a concentration of 300 ppm. The analysis of the effectiveness of HClO dry mist disinfection against this virus also showed the virucidal nature of the process at higher concentrations of HClO, i.e., 500 ppm and 2000 ppm. The reduction in other viruses subjected to HClO dry mist disinfestation, defined as normative [19], was achieved at the level of ≥4 lg required by the PN-NE 17272:2020 [18] standard at concentrations of 500 and 2000 ppm (Table 4). 

The fungicidal effect of dry mist HClO at a concentration of 300 ppm was observed against Candida albicans with a reduction of 5.71 lg. 

### 2.4. Colorimetric Measurements of Pulse Oximeter Cases

The results of colorimetric measurements of pulse oximeter case materials are shown below. The diffuse and specular reflectance spectra were measured using an integration sphere. The spectra were then converted to the CIELAB color space. The “L” value represents perceptual color lightness, where value *a* represents a red–green hue and value *b* represents a blue–yellow hue.

**Figure 2:**
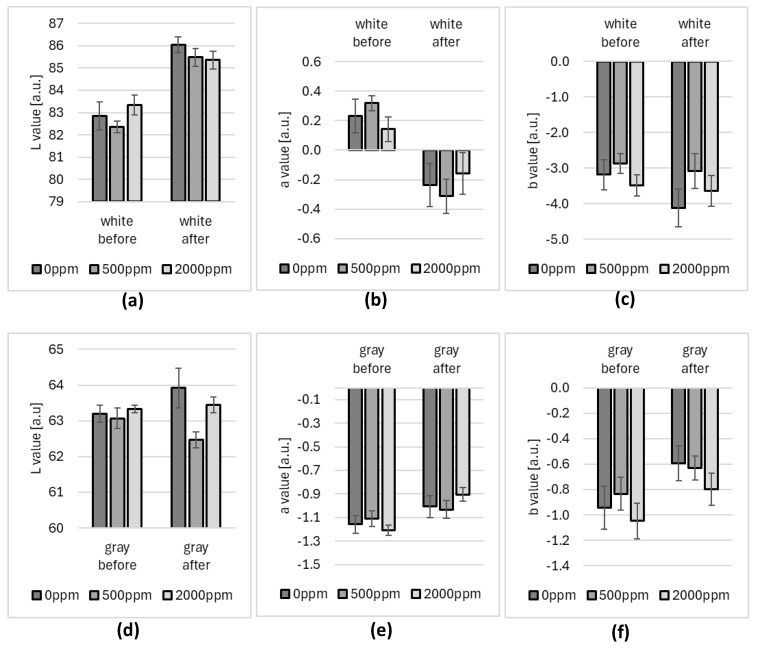
Comparison of the L, a, and b color measurement results for pulse oximeter white plastic cases (**a**–**c**) and gray plastic hinges (**d**–**f**) after 30 cycles of various concentrations of hypochlorous acid fogging (Variant II) (0 ppm means clear water fogging).

**Figure 3:**
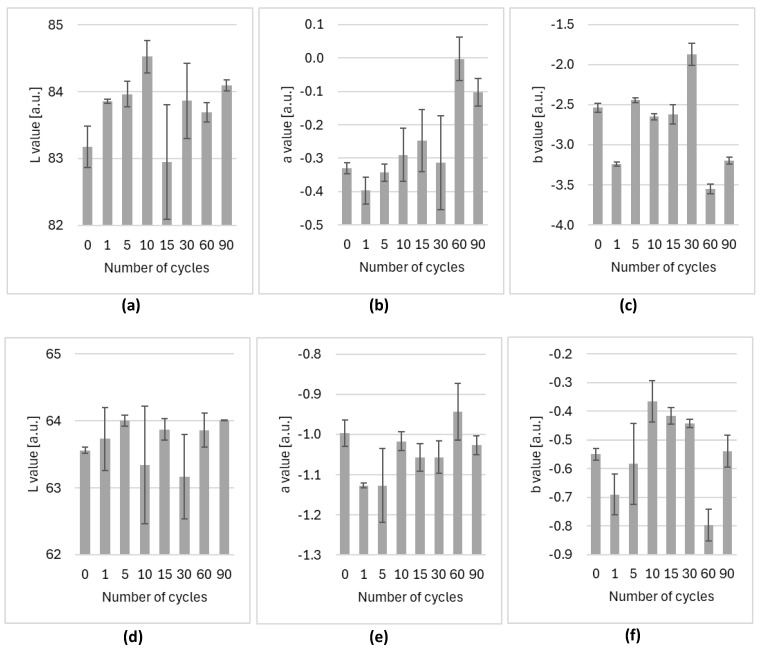
Comparison of the L, a, b color measurement results for pulse oximeter white plastic case (**a**–**c**) and gray plastic hinges (**d**–**f**) after 1 to 90 cycles of 500 ppm concentration of hypochlorous acid fogging (Variant III).

The values measured for Variant II fogging are presented in Figure 2 and the values collected for Variant III are presented in Figure 3. The error bars represent the standard deviation of values measured for five samples at each fogging concentration or cycle.

The color measurements of the pulse oximeter cases were conducted as shown in Figure 3b. The first place was white plastic on the side of the device (Figure 2a–c and Figure 3a–c), and the second place was the gray plastic connector (Figure 2d–f and Figure 3d–f). 

Figure 2 presents results after 30 cycles of fogging (Variant II) for 500 ppm and 2000 ppm concentrations of hypochlorous acid. Those results were compared to clear water fogging (marked as 0 ppm on the plots).

Figure 3 presents pulse oximeter case colors after 1, 5, 10, 15, 30, 60, and 90 cycles of fogging (Variant III). The results are compared to colors before the fogging process (marked as 0 cycles on the plots). 

The analysis of the results shows that there are slight changes in the color of the samples before and after fogging treatment (Variant II). The changes in hue (CIELAB color space a and b values) are small (differences of less than 1 unit). Those differences are imperceptible to humans and are also close to the measurement limit of the spectrometer (0.5 unit).

The most distinct change in color after treatment is visible in the lightness (L value) of white case plastic after Variant II of the fogging process. In this case, the difference is the same for the control sample (0 ppm) and test samples. This effect may be caused by the cleaning effect of fogging with water, which may make the plastic more reflective.

The changes observed during consecutive cycles of Variant III of the fogging process are mostly stochastic and lower than the spectrometer limit (0.5 unit). The only significant change is for the b channel (representing a blue–yellow hue) in the white plastic (Figure 2c). The largest change occurs between 30 and 60 cycles of fogging. Still, the change is not perceptible to the naked eye (around 1.5 units) and the plastic did not show any visual degradation.

### 2.5. Surface Tests of the Electronic Pulse Oximeter Module

**Figure 4:**
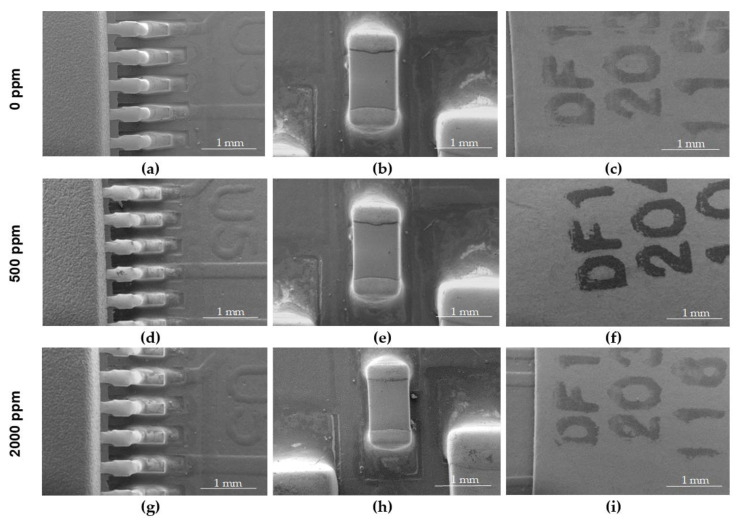
Pulse oximeter fragments subjected to SEM tests: non-fogged sample (**a**–**c**); sample fogging according to Variant II 30 cycles with hypochlorous acid at a concentration of 500 ppm (**d**–**f**); and sample fogged according to Variant II 30 cycles with hypochlorous acid at a concentration of 2000 ppm (**g**–**i**).

**Figure 5:**
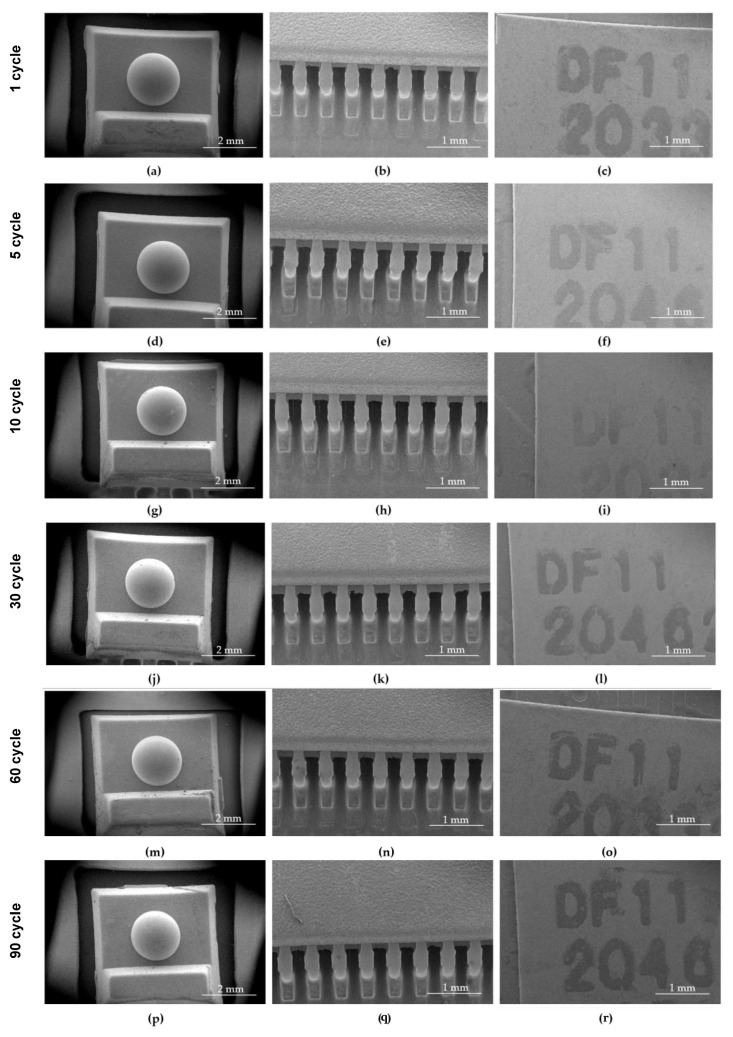
SEM images of pulse oximeter fragments subjected to the second fogging scheme: fogging sample with 1 cycle (**a**–**c**) and 5 (**d**–**f**), 10 (**g**–**i**), 30 (**j**–**l**), 60 (**m**–**o**), and 90 cycles (**p**–**r**) with HClO using a concentration of 500 ppm.

In order to determine the possibility of hypochlorous acid dry fog penetration through the casing and deposition in the form of hypochlorite crystals, an analysis of the surface of the pulse oximeter microcontroller boards was performed by a scanning electron microscope of the internal elements of the electronic module after Variant II and III fogging cycles (Figure 4 and Figure 5).

Tests performed using a scanning electron microscope with different magnifications did not reveal the presence of hypochlorous acid crystals on the surface of the internal components of the portable Microlife OXY 300 finger pulse oximeters.

### 2.6. Electronic Circuit Performance Tests

#### 2.6.1. Stress Test

**Figure 6:**
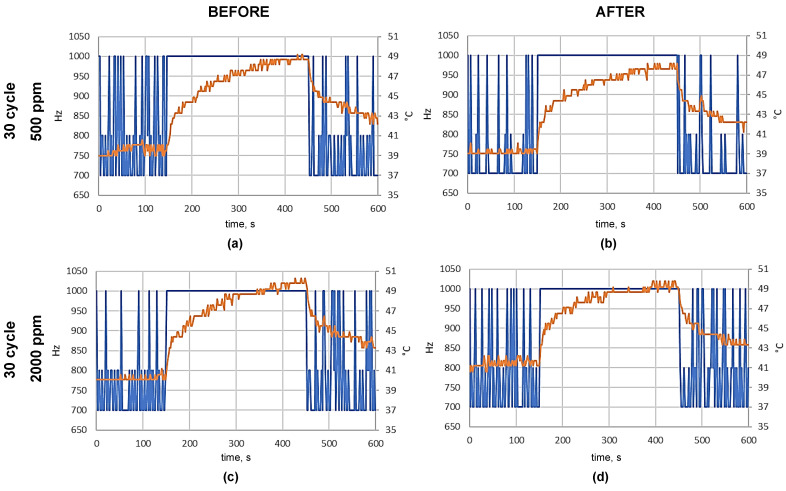
Temperature (red line) and frequency (blue line) of CPU before (**a**,**c**) and after 30 hypochlorous acid fogging cycles. The concentrations used were 500 ppm (**a**,**b**) and 2000 ppm (**c**,**d**).

**Figure 7:**
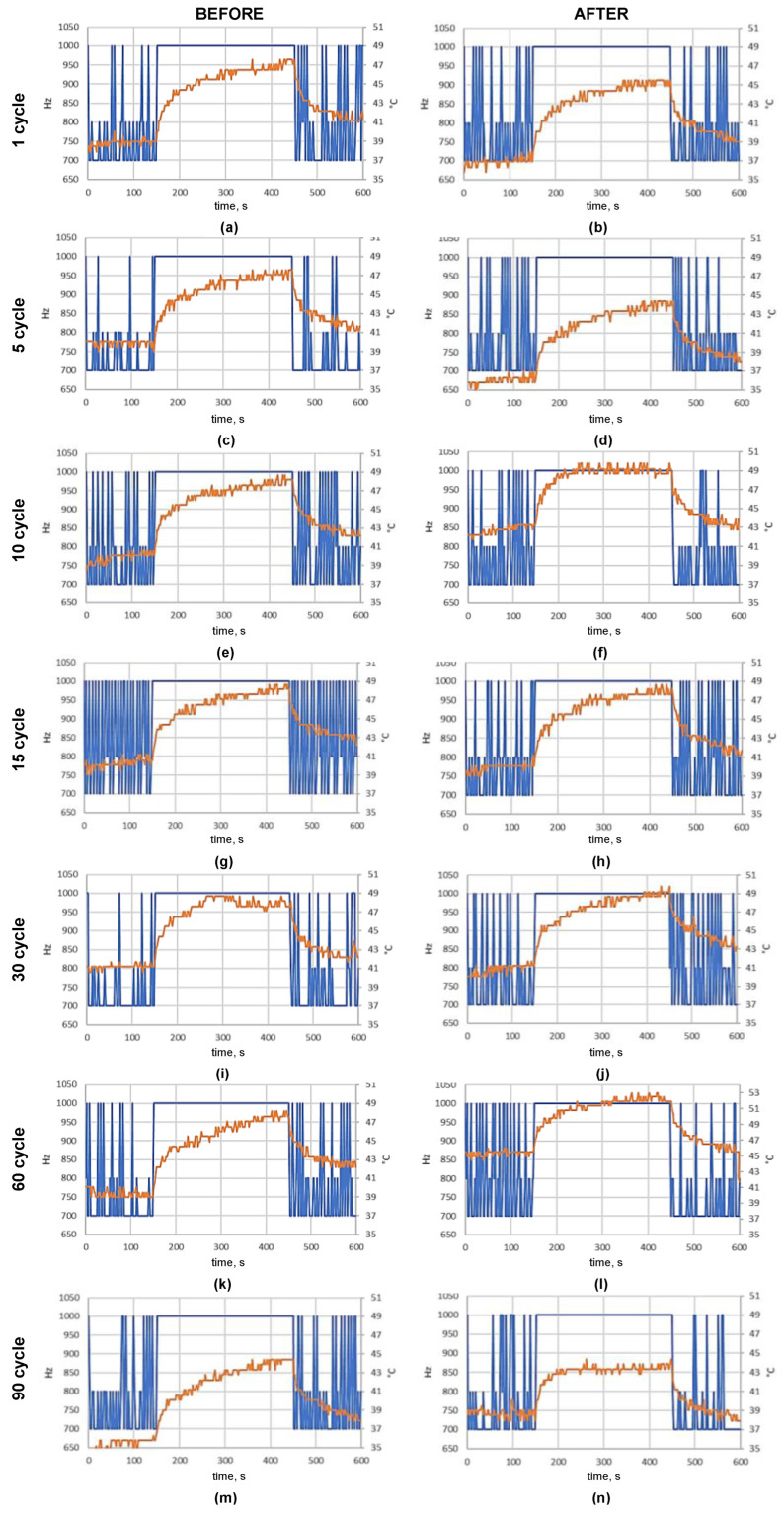
Temperature (red line) and frequency (blue line) of CPU before fogging (**a**,**c**,**e**,**g**,**i**,**k**,**m**) and after 1 (**a**,**b**), 5 (**c**,**d**), 10 (**e**,**f**), 15 (**g**,**h**), 30 (**i**,**j**), 60 (**k**,**l**), and 90 (**m**,**n**) fogging cycles with 2000 ppm of hypochlorous acid.

The Stressberry test allows the CPU temperature to be tested under different loads. The whole process takes 10 min. After 3 min of running idle, the frequency achieves a maximum level of 5 min after which it cools down. The relationship between frequency and temperature changes over time and was saved in a .csv file. According to Raspberry Pi specifications, the working temperatures were −40–85 °C. The temperature above the upper limit causes the RPi processor to slow down and cool [19]. According to the averaged operational characteristics of Raspberry Pi Zero W presented in Figure 6 and Figure 7, the average temperature did not exceed 50 °C when tested in room conditions.

#### 2.6.2. Memtester

The memory test performed on the Raspberry Pi Zero W board was intended to show any possible memory faults that could appear due to fogging in the chamber. The final result of the test was a list of checked items assigned as *OK* if the test was successfully completed or *FAILURE* alert in the case of an error. All boards successfully passed the memory tests before and after treatment with water and hypochlorous acid fog. The studies showed that the memory section can withstand the treatment with water or hypochlorous acid fog up to at least 2000 ppm for 30 cycles under the conditions set. The results are presented in Table 5.

#### 2.6.3. PWM Signal Quality

There were no noticeable differences observed between the recorded signals. Similarly, no changes were observed in the period/frequency measured and their standard deviation when a comparison was made between the PWM generated on Raspberry Pi’s GPIO pins before and after 30 fogging cycles with 2000 ppm of HClO. It can be concluded that the fogging process had no influence on the quality of PWM signal generation. 

## 3. Discussion

Hypochlorous acid can penetrate the bacterial membrane and damage its structure, leading to its destabilization and breakdown. Bacterial spores are more resistant to HClO treatment than vegetative cells, remain in their rigid structure, and show no trace of rupture or damage [10,14]. Therefore, different concentrations of hypochlorous acid and exposure times were used in the course of the research (300, 500, and 2000 ppm), as the effective application of hypochlorous acid depends on the parameters used.

As with bacteria, hypochlorous acid can attack and damage viral cells. The broad-spectrum virucidal effect of HClO is mainly due to its oxidative capacity and denaturation of viral proteins [15]. HClO can attack and damage the genetic material of the virus, i.e., RNA or DNA, to form chloramines and nitrogenous free radicals, which leads to the disruption of single- and double-stranded nucleic acids [16].

The biocidal activity assumed in the PN-EN 17272:2020 [18] standard, expressed as lg reduction at the level of ≥4 lg, was achieved for HClO in a concentration range of 300𢀓2000 ppm.

In the group of bacteria, the desired logarithmic reduction value of ≥5 lg was achieved for the HClO concentration of 300 ppm and a single fogging cycle lasting 60 min. This result was the same for the species studied in this group.

The exception was the *Clostridioides difficile* strain, for which the required reduction level ≥ 5 lg according to the PN-EN 17272:2020 standard was achieved at a higher concentration of 500 ppm.

For estimation of the sporicidal activity, the HClO dry fog *Bacillus subtilis* strain was used. Spores are very resistant to various external factors, including disinfectants. It is, therefore, not surprising that a satisfactory reduction of ≥4 lg was achieved after the application of HClO at a concentration of 2000 ppm. For additional bacterial strains, the required reduction of ≥5 lg was already achieved at a concentration of 300 ppm, and for higher concentrations (2000 ppm) the reduction reached ≥4 lg. The hypochlorous acid tested has bactericidal properties for both Gram-negative and Gram-positive bacteria.

In the group of viruses, a satisfactory result of ≥4 lg was already achieved for the modified vaccinia virus *Ankara* (MVA) as a surrogate for SARS-CoV-2 with the use of hypochlorous acid at a concentration of 300 ppm for 60 min. Therefore, this also proves the effectiveness of HClO against this virus for hypochlorous acid at higher concentrations, i.e., 500 ppm and 2000 ppm. The reduction in other viruses, defined as normative according to [18], was only achieved at the required level of ≥4 lg for hypochlorous acid at concentrations of 500 and 2000 ppm (Table 4).

In the group of fungi, sensitivity to the lowest tested acid concentration was shown by *Candida albicans* with a reduction of 5.71 lg, while *Aspergillus brasiliensis* only achieved the assumed reduction for 2000 ppm and at an extended exposure time of up to 90 min.

In the test determining the activity of acid on *Mycobacterium terrae* and *Mycobacterium avium*, the required reduction of ≥4 lg was obtained with a concentration of 500 ppm.

Studies have shown that by selecting the right concentration of hypochlorous acid dry mist, a wide spectrum of microorganisms can be inactivated: bacteria, viruses, and fungi. Many of them can be neutralized in conditions that are safe for humans. Measurements of free chlorine levels in the foggy room confirmed the safety of using hypochlorous acid at concentrations of 300 ppm and 500 ppm. At these concentrations, chlorine levels did not exceed 0.51 ppm (1.5 mg/m^3^ the lowest permissible concentration of chlorine). Only when HClO 2000 ppm was used was the free chlorine level found to be higher than the permissible level (Table 2).

Experiments with biological material were carried out for only one cycle, so the achieved effect is even better when we consider the cyclical nature of the decontamination process. The use of Variants II and III for fogging will allow for effective decontamination of medical devices used in everyday emergency medical practice and, for example, interiors of medical vehicles at a lower concentration HClO level (300 and 500 ppm) without exceeding the level of chlorine concentration that is safe for humans.

Simulations of the impact of hypochlorous acid on small medical equipment were carried out using pulse oximeters and Raspberry Pi Zero microcontrollers.

Analysis of Microlife OXY 300 finger pulse oximeter results after 30 cycles of fogging (4.5 min dry mist generation + 60 min exposure + 15 min chamber purification) showed that the changes in the shade of the samples are very small (differences of less than 1 unit). The changes are imperceptible to humans and at the same time very close to the spectrometer’s measurement limit (0.5 units). 

It was also observed that the largest changes in L-space (brightness) were caused by water fogging. One possible explanation for this phenomenon may be the cleaning properties of the water aerosol, resulting in a higher reflectivity of the samples tested. 

In addition, during colorimetric tests of pulse oximeters after 1, 5, 10, 15, 30, 60, and 90 fogging cycles (4.5 min dry fog generation + 9 min exposure + 15 min chamber cleaning), no correlation was observed between the results and subsequent fogging. Therefore, it can be concluded that hypochlorous acid mist will not cause damage to small medical equipment or shorten its usefulness. Both its aesthetic value and operating parameters will be preserved. 

Tests performed using a scanning electron microscope did not show the penetration of hypochlorous acid dry fog into the interior of the Microlife OXY 300 finger pulse oximeters. 

It is also worth emphasizing that all Raspberry Pi Zero boards tested, despite direct (without housing) exposure to hypochlorous acid, retained full functionality concerning all cycles, times, and concentrations.

## 4. Materials and Methods

Studies of the direct impact of hypochlorous acid dry fog (Section 2.1) were performed on biological material (bacteria, viruses, fungi, and spores) using Raspberry Pi Zero W microcontrollers and Mictolife OXY 300 finger pulse oximeters.

### 4.1. Fogging Process

In the course of the research, three models of fogging cycles were used (Table 6).

**Figure 8:**
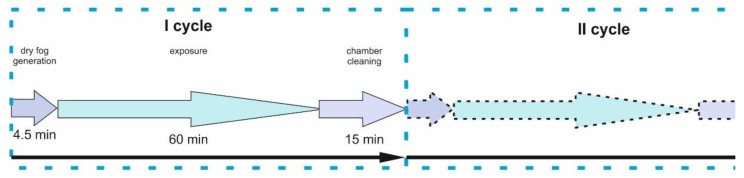
Diagram of Variant II fogging model: 30 cycles × 4.5 min of dry mist generation (25 mL/1 m^3^) + 60 min of hypochlorous acid exposure + 15 min of chamber cleaning.

**Figure 9:**
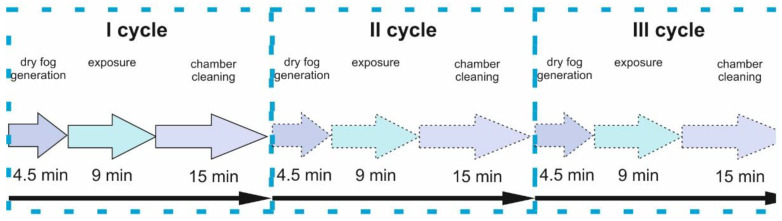
Diagram of the Variant III fogging model of 1, 5, 10, 30, 60, and 90 cycles × 4.5 min of dry mist generation (25 mL/1 m^3^) + 9 min of hypochlorous acid exposure + 15 min of chamber cleaning.

**Figure 10:**
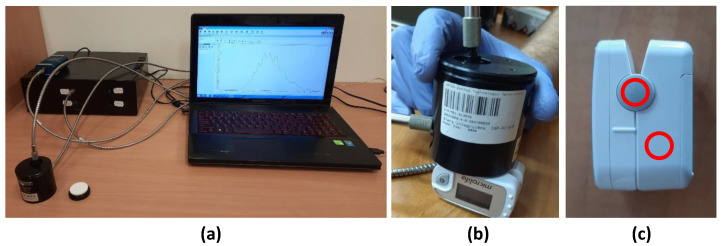
Color measurement system (**a**,**b**); measurement locations on pulse oximeter housings (**c**).

- Variant I: The simulation of a single fogging cycle in which pathogens are inactivated was carried out. This cycle was carried out in accordance with [18] and only for biological studies to simulate a one-time dry mist decontamination process of hypochlorous acid to viruses, bacteria, spores, and fungi. The distance between the dry mist nozzle and the test media was 2.6 m. The dimensions of the room in which the tests were carried out were 3.420 × 3.500 × 2.508 m. The carriers used to perform the tests were discs made of stainless steel with a diameter of 40 mm, class 2B. The disinfection process according to Variant I was carried out by generating dry mist at concentrations of 300 and 500 ppm, respectively, with a consumption of 20 mL/m^3^ of product and a contact time of 60 min, and for a concentration of 2000 ppm, 25 mL/m^3^, and a contact time of 90 min.
- Variant II: The simulation of long exposure times of a disinfectant was carried out to obtain the possibility of full decontamination of rooms, surfaces, equipment, or devices, mainly from pathogens, including drug-resistant strains. This is particularly relevant in the medical field, where antibiotic resistance of many bacteria is becoming a health and epidemiological problem. This process consisted of a total of 30 cycles × 4.5 min of dry mist generation (25 mL/m^3^) + 60 min of hypochlorous acid exposure + 15 min of chamber purification (Figure 8). The test results for this model were compared to elements fogged with pure water.

- Variant III: The simulation of HClO fogging with short exposure times and systematic removal and examination of samples after 1, 5, 10, 15, 30, 60, and 90 cycles was carried out (Figure 9). The exposure time of hypochlorous acid was 9 min. The dry mist generation (25 mL/1 m^3^) and chamber cleaning (Figure 10) times were the same as in the case of Variant II, namely 4.5 and 15 min. In Variant III fogging (Figure 9), the short times correspond to the exposure and decontamination process. At each stage, 3–5 samples were used for testing, consisting of about 40 samples in total. The results of this study were compared to elements that had not been fogged.

In the case of microbiological tests (Variant I), the process of exposure to dry mist of hypochlorous acid was carried out in the room in accordance with the standard [18]. In the case of electronic systems (Variants II and III), the process was carried out in the chamber.

The tests were carried out using a pneumatic fogger and nozzle that produced fog with a droplet size of less than 10 microns, manufactured by JFC (Karpin, Poland). 

During the tests, the free chlorine level was measured using three portable chlorine detectors from DRAGER Pac 8000 (Drägerwerk AG & Co. KGaA, Lübeck, Germany) in order to eliminate the measurement error.

During this study, the size distribution of the aerosol particles was measured using a TROTEC PC200 sensor, and the flow was 2.83 L/min (0.1 ft ³/min) controlled by an internal pump. The outlet hole sizes were as follows: 0.3 μm, 0.5 μm, 1.0 μm, 2.5 μm, 5.0 μm, and 10.0 μm. Light source: Class 3B laser, 780 nm, 90 mW.

Temperature and humidity during fogging were measured using an IST002 meter, Model HTC-1 (Meteo-logic).

### 4.2. Materials

#### 4.2.1. Hypochlorous Acid

The tests were performed using ultrapure stable hypochlorous acid with a concentration of 300, 500, and 2000 ppm (0.03, 0.05, and 0.2%), produced by BioMedAqua Sp. z o.o. (BioMedAqua Sp. z o.o., Dębica, Poland). The biocidal product marketing authorization number was in accordance with safety data sheet 8931/22.

Hypochlorous acid was synthesized by electrolysis of the solution according to Formulas (1) and (2) as follows:

2Cl^−^+ 2e^−^→ Cl_2_ (1)

Cl_2_ + H_2_O → HClO + H^+^ + Cl^−^ (2)

#### 4.2.2. Biological Material

Studies of the effect of HClO dry mist on the selected biological material included the following strains:

- Bacteria: Escherichia coli, Acinetobacter baumannii, Pseudomonas aeruginosa, Proteus hauseri, Enterococcus hirae, Salmonella Typhimurium, Salmonella Enteritidis, Listeria monocytogenes, Staphylococcus aureus, Legionella pneumophila, Mycobacterium avium, Mycobacterium terrae, and Clostridioides difficile.
- Bacterial spores: Bacillus subtilis.
- Viruses: Murine Norovirus, Adenovirus type 5, Porcine Parvovirus, and Modified vaccinia virus Ankara (MVA).
- Fungi: Aspergillus brasiliensis and Candida albicans.

These strains were purchased from the American Type Culture Collection (ATCC) and the Friedrich Loeffler Institute. Although the tests were carried out in accordance with the procedure found in the PN-EN 17272:2020 standard [18], in order to fully confirm the effectiveness of HClO dry mist exposure, a larger number of strains were used than assumed by the above-mentioned standard [18]. Additional microorganisms include *Salmonella enterica* subsp. *enterica serovar Typhimurium*, *Salmonella enterica* subsp. *enterica serovar Enteritidis*, *Listeria monocytogenes*, *Clostridioides difficile*, *Legionella pneumophla*, and modified vaccinia virus Ankara (MVA). The incubation conditions for strains of bacteria, viruses, spores, and fungi, along with the type of medium, were in accordance with the recommendations of the PN-EN 17272:2020 standard [18] and detailed data are included in Table 7 and Table 8. 

The diluent for microbial suspension (liquid for test organism recovery and rinsing liquid for membrane filtration)—consisting of tryptone (OXOID/Thermo Fisher, Waltham, MA, USA), pancreatin casein hydrolyzate (1.0 g/*OXOID*/Thermo Fisher, Waltham, MA, USA), sodium chloride (8.5 g/BTL Sp. z o.o. Enzyme and peptone plant, Łódź, Poland), and freshly distilled water in a glass apparatus (1000 mL)—was sterilized in an autoclave at 121 °C for 15 min. 

Bovine albumin (Cohn V fraction) (Merck Life Science, Rahway, NJ, USA) was used as an interfering substance at a concentration of 0.3 g of bovine albumin (Roche Diagnostics International AG, Rotkreuz, Switzerland)/100 mL of distilled water. The sterilization process was carried out using filtration through a membrane filter (Merck Life Science, Rahway, NJ, USA) with a micro-hole size of 0.22 μm. 

#### 4.2.3. Portable Pulse Oximeters

Tests using Microlife OXY 300 portable finger pulse oximeters (Microlife AG, Widnau, Switzerland) included an analysis of the change in the reflectance spectrum (color) of the case and the effect of dry fog on internal components before and after fogging processes. A total of 70 pieces of the oximeters were used in this study.

#### 4.2.4. Raspberry Pi Zero Computer Boards

Raspberry Pi Zero W computer boards were used to mimic the possible influence of hypochlorous acid on electronic circuits and possible corrosion that can take place in medical devices caused by fogging with hypochlorous acid. Raspberry Pi Zero is a single-board computer (SBC) with a BCM2835 1 GHz single-core processor and 512 MB of RAM. Its size is 66.0 mm × 30.5 mm × 5.0 mm and it weighs only 9 g. The board is equipped with 40 GPIO pins. The OS Debian version 11 (bullseye) was downloaded and run using a 16 MB SD Card. The 54 units of Raspberry Pi Zero boards were tested in the project.

### 4.3. Experimental Methods

#### 4.3.1. Physicochemical Tests of Hypochlorous Acid

Tests of the measurement of the surface tension of hypochlorous acid were carried out using the bubble method to measure the pressure necessary to push the air bubble out of the tube immersed in the liquid tested (hypochlorous acid). 

The chemical structure of hypochlorous acid was confirmed by FTIR spectroscopy (Nicolet IS50, FTIR, ThermoFisher SCIENTIFIC, Waltham, MA, USA). The presence of characteristic absorption bands was recorded. Samples were measured using the ATR technique (Attenuated Total Reflection) in a range of 400–4000 cm^−1^ with a resolution of 4 cm^−1^, and the number of scans was 64.

#### 4.3.2. Studies on the Impact of Dry Mist HClO on Selected Bacteria, Viruses, Spores, and Fungi

To evaluate the effectiveness of the automated disinfection process, tests were carried out according to the conditions specified in the standard [18] with the use of hypochlorous acid with concentrations of 300 ppm, 500 ppm, and 2000 ppm in the form of dry mist. The tests measured the reduction (expressed as a decimal logarithm (lg)) in the number of surviving organisms of different strains of bacteria, viruses, spores, and fungi under specific conditions.

#### 4.3.3. Bacterial Cultures and Bacterial Spores

Working cultures of bacteria from baseline cultures made from reference strains were prepared (Table 9). Then, suspensions of microorganisms with the required inoculum of 5 × 10^7^–2 × 10^9^ CFU/mL were prepared based on [18]. Bovine serum (interfering substance) was added to the suspension 1:10 (*v*/*v*). 

Murine Norovirus S99 Berlin, Adenovirus type 5 (AV-5) ATCC VR, *Porcine parvovirus* (PPV) ATCC VR-742, and modified vaccinia virus Ankara (MVA) ATCC VR-1566 were used in these experiments. They were cultivated using the following cell lines: RAW 264.7 ATCC TIB-71for MVM, ST cells ATCC CR-1746 for PPV, HeLa cells ATCC CCL-2 for AV-6, and BHK-21 ATCC CCL-10 for MVA. Cells were cultivated in the media presented in Table 10 with a dedicated concentration of fetal bovine serum (FBS) and penicillin/streptomycin at 37 °C + 5% CO_2_.

Virus suspensions for testing should have a TCID50 (cell culture infective dose, defined as the virus dilution required to infect 50% of cells) of 1 × 10^7^–1 × 10^9^/mL [18]. The monolayer microplate culture method was used to determine the virus titer, determining the viral cytopathic effect observed using an inverted microscope (CKX 53 with the U-TV0 Olympus adapter). Bovine serum (interfering substance) was added to the suspension at a ratio of 1:10 (*v*/*v*). 

#### 4.3.4. Fungal Cultures

The evaluation of Aspergillus brasiliensis spores with a titer of 5 × 10^6^–1 × 10^7^ CFU/mL needed to make a suspension was performed in macroscopic analysis at 400-fold magnification. A total of 75% of spiny spores were observed, with no signs of germination and no mycelium fragments, which indicates that the suspension was suitable for this study and was in accordance with the recommendation of the PN-EN 17272:2020 standard [18].

#### 4.3.5. Preparation of Carriers for Assessment of Hypochloric Acid Effect on Bacteria Cultures, Viruses, Spores, and Fungi for Carrying out the Effectiveness Test

In the evaluation of the efficacy of hypochlorous acid against selected strains of bacteria, viruses, spores, and fungi, the carriers in the efficacy test were in a vertical position at a height of 1.5 m from the floor, while the inoculum with a dedicated concentration for a given microorganism was turned away from the source of HClO distribution.

Suspensions of microorganisms in the form of one drop (50 μL) were applied to the center of a steel carrier placed on sterile Petri dishes. The inoculum was then evenly distributed over the entire surface of the carrier. A sterile inoculating loop was used to evenly cover an area with a diameter of 2 ± 0.2 cm^2^. The carriers were placed on an open Petri dish and dried for <120 min at a temperature below 37 °C. The procedure of contamination of carriers with microorganisms was in accordance with the recommendations of the PN-EN 17272:2020 standard [18]. Three-fold replicates of the carrier contamination were performed for each microorganism strain tested.

After drying, a hypochlorous acid dry mist was generated. The control group consisted of carriers contaminated with microorganism cultures not exposed to dry mist HClO. The storage conditions (temperature and humidity) of the control media carriers were identical to those of the carriers disinfected with HClO dry mist. During the process, they were stored at temperatures and humidity similar to those recorded in the room where the disinfection process was carried out.

After the disinfection process was completed, each carrier was transferred to flasks containing 50 mL of sterile recovery liquid and glass beads to test the effectiveness of dry mist hypochlorous acid on microorganisms. They were then shaken for a few seconds manually and for 1 min on a rotary shaker (SKO-0330-PRO ChemLand, Stargard, Poland) to remove the test organisms from the carriers. Decimal dilutions were then made. From the dilutions, a pour plate of 1 mL was made from 3 consecutive dilutions in 2 replicates. 

In the case of bacterial Mycobacterium and Legionella pneumophila, a surface culture of 1 mL divided into two equal volumes was dispensed simultaneously on two plates. The remaining recovery fluid was plated by membrane filtration in a volume of 10 mL per plate and the residue on the other plate. After filtration of the appropriate volume, the filters were rinsed with 50 mL of recovery fluid. The membrane filters were then transferred to suitable agar media. Each of the carriers was removed aseptically from the flask and placed on an upward-facing inoculum Petri dish. The medium was poured with an appropriate amount of nutrient solution to cover the carrier evenly. In the case of *Mycobacterium terrae*, *Mycobacterium avium,* and *Legionella pneumophila*, the carriers were removed aseptically from the flask and placed on an agar medium. Incubation was carried out in accordance with the conditions set out in Table 9 and Table 10.

For the additional strains, *Salmonella enterica* subsp. *enterica serovar Typhimurium*, *Salmonella enterica* subsp. *enterica serovar Enteritidis*, *Listeria monocytogenes*, and *Clostridioides difficile*, the procedures were the same as for the other bacteria. In the case *of Legionella pneumophla,* inoculum was prepared the same as for the other bacteria, and the evaluation of bactericidal activity was performed the same as for *Mycobacterium terrae* and *Mycobacterium avium*. 

To determine the virucidal effect after the disinfection process, the carriers were transferred to flasks with 20 mL of recovery liquid and glass beads in accordance with the guidelines of PN/EN 17272:2020 [18]. The flasks were stirred by hand for a few seconds and decimal dilutions were made in a culture medium suitable for the cell line (Table 3 and Table 5). An amount of 0.1 mL of each dilution was transferred to an 8-well microplate with a single-layer cell culture of cell lines dedicated to a given virus strain. After 1 h of incubation at 37 ± 1 °C, 0.1 mL of cell culture was added and incubated according to Table 9 and Table 10. The viral cytopathic effect was analyzed using an inverted microscope (CKX 53 with Olympus U-TV0 adapter).

#### 4.3.6. Effect of Hypochlorous Acid on the Level of Logarithmic Reduction

For each strain of bacteria, fungi, and the carrier tested from the disinfected group and from the control group, the number of colonies grown on Petri dishes and/or filters (n′_1_, n′_2_ for test samples; n_1_, n_2_ for control carriers) was determined according to Formulas (3)–(5). 

In order to calculate the mean logarithmic reduction for virucidal activity, lgTCID50 calculations were made for the initial viral load, the result for the control carrier, and the result for the test carrier using the Spearman–Kärber method [20].

The reduction lg (R) was calculated from the difference between the control media carriers’ logarithmic titer and the carriers subjected to HClO dry mist disinfection. 

In order to calculate the logarithmic reduction (R) (3) for the remaining microorganisms, the number of test organisms in the starting suspension (N) (4), the number of test organisms obtained on control carriers not exposed to dry mist HClO (T) (5), and the number of organisms on the test carriers were first calculated as follows:

(3) $$R=lg\frac{T}{\left({n^{\prime }}_{1}+{n^{\prime }}_{2}\right)}$$

where

n′_1_—the number of surviving test organisms.
n′_2_—the number of colonies obtained by deep culture of the test vehicle.

After obtaining the reduction, the mean was calculated for each of the 3 test carriers as follows:

(4) $$N=\frac{c}{\left(n_{1}+0.1n_{2}\right)}\times d\times V\;\frac{CFU}{ml}$$

(5) $$T=\frac{c}{\left(n_{1}+0.1n_{2}\right)}\times d\times V\times 100\;\frac{CFU}{ml}$$

where

c—the sum of CFU counts on all included plates.
n_1_—the number of platelets considered in the lower dilution.
n_2_—the number of platelets considered in the higher dilution.
V—the sample volume.
d—the dilution factor corresponding to the lower dilution.

#### 4.3.7. Colorimetric Tests of Pulse Oximeter Plastic Cases

Tests of medical–diagnostic devices included analysis of the change in the reflectance spectrum (color) of the housing and a check on the effect of dry fog on internal elements before and after the fogging processes. In accordance with the objectives of the project, the tests were carried out on a selected type of medical–diagnostic device, namely pulse oximeters (Figure 10). 

Colorimetric measurements were made using the AvaSpec-ULS2048L-2-USB2 spectrometer from Avantes (Apeldoorn, the Netherlands) equipped with an ISP-30-6-R integrating sphere from Ocean Optics (Ostfildern, Germany). The integrating sphere allows the measurement of all reflected and scattered radiation on the sample. The samples were illuminated with a halogen source LS-1 (Ocean Optics). The white standard USRS99-010 reference from Labsphere (North Sutton, NH, USA) was used to calibrate the instrument.

The colorimetric coordinates in the CIELAB system were calculated using AvaSoft software from Avantes. The CIELAB colorimetric space describes the color using three components: L—brightness (0–100); a—green (−100)–magenta (+100); and b—blue (−100)–yellow (+100). Color differences of less than 2 units are imperceptible to the average observer. The perceptual total color difference between the samples in the CIELAB color space is calculated as the Euclidean distance between two points represented by the following lab coordinates:

(6) $$∆E=\sqrt{{\left(L_{1}-L_{2}\right)}^{2}+{\left(a_{1}-a_{2}\right)}^{2}+{\left(b_{1}-b_{2}\right)}^{2}}$$

Color measurements were made in two places of the pulse oximeter housing: on the flat surface of the white plastic and in the middle of the gray hinge connecting the two parts of the housing.

#### 4.3.8. Surface Testing of the Electronic Module of the Finger Pulse Oximeters

An analysis of the internal elements of the pulse oximeter microcontroller plates after the given fogging cycles according to the II and III Variants was performed (Figure 8 and Figure 9). The structural assessment of the surface of samples subjected to hypochlorous acid fogging was performed using the Quanta 250 FEG scanning electron microscope (Quanta 250 FEG SEM, FEI, Hillsboro, OR, USA). An SEM image was created with a distributed detector (ETD-BSE, FEI, Hillsboro, OR, USA) with an acceleration voltage of 10 kV. In order to improve the conductivity of the tested wafer surfaces, sputtering of a 5.18 nm gold layer was performed using an EM ACE 600 high-vacuum sputterer.

#### 4.3.9. Performance Tests of Raspberry Pi Zero Electronic Circuit Microcontrollers

Three different tests were performed to compare the results and determine if hypochlorous acid fogging had an impact on the performance of the RPi [21,22,23,24]. The boards were tested before and after hypochlorous acid treatment.

**Figure 11:**
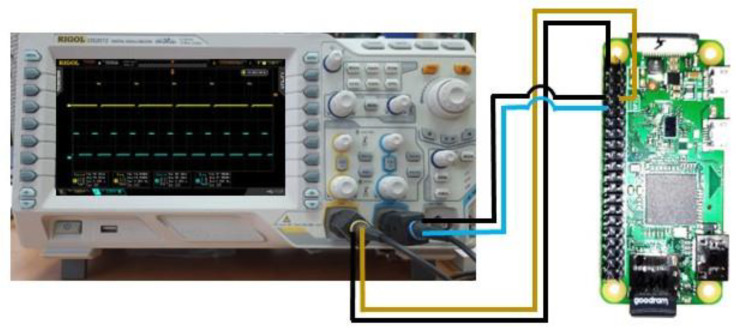
Schematic diagram of Raspberry Pi Zero PWM signal quality testing.

- Memory test (sudo apt-get install memtester v4.5.0 (32-bit)). This tests the stability of the RAM. It effectively performs stress-testing of the memory subsystem and helps to find intermittent and non-deterministic faults [24].
- StressTest (pip install StressberryStress v0.3.3). The Stressberry test runs a series of processes on the system. It is designed to run the CPU at full power and monitor the temperature and stability of the system [25].
- A GPIO pin test was used to check the performance of GPIO pins. The hardware PWM signal was measured before and after treating the RPi board with hypochlorous acid fog. For the performance test, the PWM signals of different frequencies were simultaneously generated on two pins—GPIO 12 and GPIO 13—with 19 kHz and 10% duty and 38 kHz and 30% duty, respectively. The PWM signals generated with RPi were measured simultaneously with a two-channel Rigol DS2072 oscilloscope. The frequency standard deviation was calculated at about 100 signal readings. The experimental setup is shown in Figure 11.

## 5. Conclusions

Based on the results obtained, the following conclusions were made:

- For the bacterial strains studied, even the lowest concentration of acid was sufficient to reduce the colony by the desired amount of logarithmic reduction (lg);
- In the group of viruses, concentrations of 300–500 ppm had a biocidal effect in accordance with the standard [19];
- The most resistant bacterial strains turned out to be bacterial spores of *Bacillus subtilis* and the strain of *Closteridiodes difficile*, for which the biocidal dose was 2000 ppm;
- No effect of fogging on the casing of the tested medical devices was observed;
- Changes in L, a, and b before and after fogging are not statistically significant, which indicates that HClO has no effect on the external material of pulse oximeters and microcontrollers;
- Repeated fogging does not affect the aesthetic appearance of medical devices;
- No penetration of hypochlorous acid into the devices and thus no effect on electronic components was observed;
- The lack of negative impact of HClO in each tested concentration was confirmed by performance tests of the tested devices.

The test results presented prove and confirm the effective disinfecting properties of the dry mist of hypochlorous acid. This disinfectant demonstrates the potential for treatment of a wide range of bacterial, viral, and fungal agents while maintaining safe decontamination conditions for personnel and medical equipment. 

## Figures and Tables

**Table 1:** Surface tension and density of hypochlorous acid.

Hypochlorous Acid Concentration ppm	Density in 20 °C g/cm^3^	Density in 22°C g/cm^3^	Surface Tension mN/m
200 ppm	0.9989	0.9987	72.35
300 ppm	0.9988	0.9986	72.35
400 ppm	0.9989	0.9987	72.35
500 ppm	0.9988	0.9990	72.35
2000 ppm	1.0060	1.0058	71.77

**Table 2:** Summary of free chlorine levels for HClO concentrations of 300 ppm, 500 ppm, and 2000 ppm.

Chlorine Concentration Reading Time after Fogging	HClO 300 ppm	HClO 500 ppm	HClO 2000 ppm
	Sensor DRAGER Pac 8000 Serial No ARRC-1259 [ppm] Cl	Sensor DRAGER Pac 8000 Serial No ARRC-1260 [ppm] Cl	Sensor DRAGER Pac 8000 Serial No ARRC-1261 [ppm] Cl
2 min	0.15 ± 0.00	0.15 ± 0.00	1.48 ± 0.03
5 min	0.17 ± 0.02	0.20 ± 0.00	1.53 ± 0.03
10 min	0.12 ± 0.02	0.20 ± 0.00	1.47 ± 0.06

**Table 3:** Summary of the particle distribution size of hypochlorous acid dry mist aerosol for concentrations of 500 ppm.

Channel Diameter μm	Aerosol Particle Diameter before Experiments μm	Aerosol Particle Diameter after 4.5 min of Dry Fog Generation, 500 ppm
0.3	2446 ± 872	35,401 ± 2152
0.5	1016 ± 350	24,269 ± 3507
1.0	116 ± 68	4448 ± 482
2.5	12 ± 9	886 ± 177
5.0	0 ± 0	99 ± 28
10.0	0 ± 0	24 ± 7

**Table 4:** Studies of the efficacy of HClO, expressed as logarithmic reduction (lg).

	Mandatory Logarithmic Reduction according to [18]	Logarithmic Reduction (lg)	Concentration of HClO [ppm]	Interaction Time with HClO
Modified vaccinia virus Ankara (MVA)—analog of SARS-CoV-2	≥4	5.50	300	60
Murine Norovirus		4.37	500	60
Adenovirus type 5		5.36	500	60
Porcine parvovirus	≥4	4.0	2000	90
*Staphylococcus aureus*	≥5	6.83	300	60
*Acinetobacter baumanii*		6.49		
*Pseudomonas aeruginosa*		6.15		
*Proteus hauserii*		6.83		
*Enterococcus hirae*		6.59		
*Escherichia coli*		6.15		
*Salmonella enterica* subsp. *enterica serovar Enteritidis*		6.66		
*Salmonella enterica* subsp. *enterica serovar Typhimurium*		6.96		
*Listeria monocytogenes*		6.9		
*Legionella pneumophila*		6.26		
*Clostridiodes difficile*	≥5	5.1	500	60
*Aspergillus brasiliensis*	≥4	5.43	2000	90
*Candida albicans*	≥4	5.71	300	60
*Mycobacterium avium*	≥4	6.12	500	60
*Mycobacterium terrae*		5.96	500	60
*Bacillus subtilis*	≥4	5.49	2000	90

**Table 5:** Memtester results show that all test procedures were successfully completed according to Variants II and III.

Item	Variant II		Variant III
	500 ppm	2000 ppm	500 ppm
Stuck Address	ok	ok	ok
Random Value	ok	ok	ok
Compare XOR	ok	ok	ok
Compare SUB	ok	ok	ok
Compare MUL	ok	ok	ok
Compare DIV	ok	ok	ok
Compare OR	ok	ok	ok
Compare AND	ok	ok	ok
Sequential Increment	ok	ok	ok
Solid Bits	ok	ok	ok
Block Sequential	ok	ok	ok
Checkerboard	ok	ok	ok
Bit Spread	ok	ok	ok
Bit Flip	ok	ok	ok
Walking Ones	ok	ok	ok
Walking Zeroes	ok	ok	ok
8-bit Writes	ok	ok	ok
16-bit Writes	ok	ok	ok

**Table 6:** Conditions of the studies conducted for individual variants and research materials.

	Object of Research	Number of Fogging Cycles	Hypochlorous Acid Concentration [ppm]	Dry Mist generation Time [min]	Dry Mist Exposure Time [min]	Amount of HClO [ml/m^3^]
Variant I	Biological material	1	300	4.5	3	20
			500		3	
			300		60	
			500		60	
			2000		90	25
Variant II	Microcontrollers Raspberry Pi Zero W and finger pulse oximeters Microlife OXY 300	30	500	4.5	60	25
			2000			
Variant III	Microcontrollers Raspberry Pi Zero W and finger pulse oximeters Microlife OXY 300	1	500	4.5	9	25
		5				
		10				
		15				
		30				
		60				
		90				

**Table 7:** List of bacterial and fungal strains used for this study and corresponding media.

Strain	Reference Number	Medium
***Staphylococcus aureus***	ATCC 6538	Tryptone Soya Agar (Graso Biotech, Starogard Gdański, Poland)
***Acinetobacter baumanii***	ATCC 19606	
***Pseudomonas aeruginosa***	ATCC 15442	
***Proteus hauserii***	ATCC 13315	
***Enterococcus hirae***	ATCC 10541	
***Escherichia coli***	ATCC 10536	
***Salmonella enterica servoar typhimurium***	ATCC 13311	
***Salmonella enterica servoar enteritidis***	ATCC 13076	
***Listeria monocytogenes***	ATCC 33152	
***Bacillus subtilis***	ATCC 6633	
***Aspergillus brasiliensis***	ATCC 19606	Malt Extract Agar (BTL Sp. z o.o. Department of Enzymes and Peptones, Łódź, Poland)
***Candida albicans***	ATCC 10321	
***Clostridiodes difficile***	ATCC 9689	Brain Heart Infusion, agar (OX OXOID/Thermo Fisher Diagnostics Sp. z o.o., Warsaw, Poland) + 0.5% yeast extract (BTL Sp. z o.o. Department of Enzymes and Peptones, Łódź, Poland) + 1% taurochlic acid (Merck Life Science Ltd., Poznań, Poland)
***Mycobacterium avium***	ATCC 15769	Middlebrook Agar with supplement (OADC)/(Becton Dickson GmbH, Heidelberg, Germany)
***Mycobacterium terrae***	ATCC 15755	
***Legionella pneumophila***	ATCC 33152	Buffered Charcoal Yeast Extract Agar (OXOID/Thermo Fisher Diagnostics Sp. z o.o., Warsaw, Poland)

**Table 8:** List of virus strains used for this study and corresponding media.

Strain	Reference Number	Cell Line	Cell Line Reference Number	Medium
***Murine Norovirus***	S99 Berlin	RAW 264.7	ATCC TIB-71	DMEM (Merck Life Science Ltd., Poznań, Poland)
***Adenovirus type 5***	ATCC VR-1516	HeLa	ATCC CCL-2	EMEM (Merck Life Science Ltd., Poznań, Poland)
***Porcine*** ***Parvovirus***	ATCC VR-742	ST	CRL-1746	
***Modified vaccinia virus Ankara (MVA)***	VR-1566	BHK-21	ATCC CCL-10	

**Table 9:** List of strains of bacteria, fungi, and corresponding media, incubation conditions, as well as the number of test microorganisms obtained in the initial suspension, and environmental parameters at the beginning of the disinfection process.

Strain	Medium	Incubation Conditions	Initial Cell Number (N) [CFU/mL]	Initial Humidity [%]	Initial Temperature [°C]
***Staphylococcus aureus***	Tryptone Soya Agar	37 °C/48 h	2.95 × 10^8^	57.2	20.9
***Acinetobacter baumanii***			1.84 × 10^9^	60.5	20.9
***Pseudomonas aeruginosa***			1.36 × 10^8^	58.3	21.6
***Proteus hauserii***			1.82 × 10^9^	60.5	20.9
***Enterococcus hirae***			8.15 × 10^8^	65.4	22.0
***Escherichia coli***			1.55 × 10^8^	58.3	21.6
***Salmonella enterica servoar typhimurium***			3.40 × 10^8^	61.4	21.8
***Salmonella enterica servoar enteritidis***			1.69 × 10^9^	61.4	21.8
***Listeria monocytogenes***			1.94 × 10^9^	65.4	22.0
***Bacillus subtilis***		37 °C/72 h	5.0 × 10^6^	58.6	20.7
***Aspergillus brasiliensis***	Malt Extract Agar	30 °C/72 h	1.0 × 10^7^	65.4	22.0
***Candida albicans***			9.95 × 10^7^	60.3	21.6
***Clostridiodes difficile***	Brain Heart Infusion, agar + 0.5% yeast extract + 1% taurocholic acid	37 °C/5 days, anaerobic conditions	7.35 × 10^8^	56.3	21.1
***Mycobacterium avium***	Middlebrook Agar z supplement (OADC)/(Becton Dickson GmbH, Heidelberg, Germany)	37 °C/21 days, increased humidity conditions	1.25 × 10^8^	64.8	21.3
***Mycobacterium terrae***			4.1 × 10^7^	64.8	21.3
***Legionella pneumophila***	Buffered Charcoal Yeast Extract Agar (OXOID/Thermo Fisher Diagnostics Sp. z o.o., Warsaw, Poland)	37 °C/5 days, increased humidity conditions	4.55 × 10^8^	60.7	21.0

**Table 10:** List of virus strains used for this study and corresponding media, incubation conditions, as well as the obtained number of test microorganisms in the initial suspension, and environmental parameters at the beginning of the disinfection process.

Strain	Cell Line	Reference Number	Medium	Incubation Conditions	Initial Suspension Titer [lgTCID_50_]	Initial Humidity [%]	Initial Temperature [°C]
***Murine Norovirus (MVM)***	RAW 264.7	ATCC TIB-71	DMEM	37 °C+5% CO_2_/up to 4 days	7.13	60.7	20.9
***Adenovirus type 5 (AV 5)***	HeLa	ATCC CCL-2	EMEM		7.5	60.7	20.9
***Porcine*** ***Parvovirus (PPV)***	ST	CRL-1746			7.13	59.9	21.9
***Modified vaccinia virus Ankara (MVA)***	BHK-21	ATCC CCL-10			7.5	60.7	20.9

## Data Availability

Data is contained within the article.
